# Validated Spectrophtometric Method for Determination of Tamsulosin in Bulk and Pharmaceutical Dosage Forms

**Published:** 2014

**Authors:** Massoud Amanlou, Amin Ghazi Moghadam, Maliheh Barazandeh Tehrani, Effat Souri

**Affiliations:** Department of Medicinal Chemistry, Faculty of Pharmacy and Drug Design and Development Research Center, Tehran University of Medical Sciences, Tehran (14155-6451), Iran.

**Keywords:** Tamsulosin, Bromocresol green, Spectrophotometry, Ion-pair complex

## Abstract

In this study a sensitive, simple and accurate spectrophotometric method was suggested for determination of tamsulosin in bulk powder and pharmaceutical dosage form based on the formation of an ion-pair complex between the drug and bromocresol green in a buffer solution at pH 3.5. The formed yellow color complex was extracted with chloroform and measured at 415 nm. The optimum reaction conditions such as pH, reagent amount, extracting solvent and the stoichiometry of the ion-pair complex were investigated. Under the optimized conditions, the Beer's law was obeyed in the concentration range of 1-160 g/mL with acceptable correlation coefficient (r^2 ^> 0.9997) and precision (CV < 3%) and accuracy (error < 2%). The proposed method was successfully used for the determination of tamsulosin in pharmaceutical capsule with nosignificant interferences of excipients.

## Introduction

**Figure 1:**
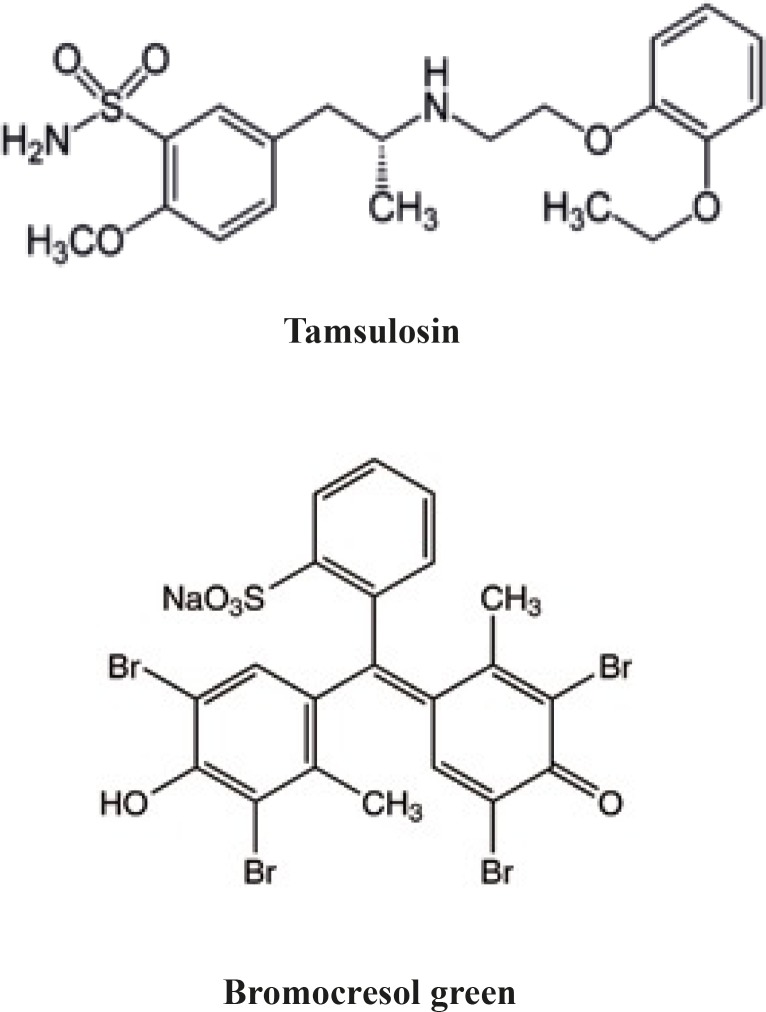
Chemical structure of tamsulosin and bromocresol green

Tamsulosin hydrochloride, (-)-(R)-5-[2-[[2-(o-ethoxyphenoxy) ethyl] amino] propyl]-2- methoxy benzene sulfonamide] (Figure 1), is a _1_ receptor antagonist, which is used for the treatment of benign prostate hyperplasia. Tamsulosin is selective for _1A _and _1D1_ subtypes and has little effect on blood pressure (1).

Several HPLC (2), LC-ESI-MS/MS (3, 4) and LC-MS/MS (5-7) methods have been reported for the determination of tamsulosin in biological fluids. Analysis of tamsulosin in bulk drug or dosage forms using HPLC (8, 9), LC-ESI-MS/MS (10), or HPTLC (11) methods reported by some authors. There are also some reports for chiral determination of tamsulosin hydrochloride (12, 13).

As the dosage form matrix is not too much complex, simple, accurate and cost effective methods with higher environmental safety are preferred for routine analysis. Recently few spectrophotometric methods were utilized for the determination of tamsulosin in the literature (14-16). 

Development of rapid and simple methods for determination of drugs in a wide range concentration is in great interest. In the continuation to our interest and previous projects to develop spectrophotometric methods for determination of some drugs (17-21), in this study a spectrophotomertic method based on ion-pair complexation utilizing bromocresol green developed for the determination of tamsulosin hydrochloride in bulk drug and dosage form.

## Experimental

*Chemicals*

Tamsulosin hydrochloride was from Ragactives (Spain). Bromocresol green (BCG) and all other chemicals were of analytical grade and purchased from Merck (Darmstadt, Germany).

*Instrument*

A double beam UV-visible spectrophotometer from Schimadzu, Japan (UV-160) was used for all experiments. Fixed bandwidth of 2 nm and quartz cuvettes with 1 cm diameter was used.

*Standard solutions*

Standard solution of tamsulosin hydrochloride was prepared by dissolving appropriate amounts of drug in distilled water to reach a concentration of 510^-4 ^M.

Calibration solutions of tamsulosin hydrochloride in the range of 1-160 g/mL were prepared by appropriate dilution of a standard stock solution (200 g/mL) by distilled water. 

Bromocresol green solution (510^-4^ M) was prepared dissolving 349 mg of BCG in 1000 mL distilled water in the presence of 2 mL of 0.1 M NaOH.

Britton- Robinson buffer solution in the pH range of 2-5 was prepared by mixing equal volumes of 0.1 M H_3_BO_3_, 0.1 M H_3_PO_4_ and 0.1 M CH_3_COOH and adjusting the pH to the desired value using 0.2 M NaOH.

Phosphate buffer (0.1 M) was prepared by dissolving appropriate amount of NaH_2_PO_4_ in distilled water and adjusting the pH value to 3.5.

Phthalate buffer (0.1 M) was prepared by dissolving appropriate amount of potassium hydrogenphthalate in distilled water and adjusting the pH value to 3.5. 

*General procedure (Sample preparation)*

Two milliliter of tamsulosin hydrochloride solution (510^-4^ M) was pipetted into a 100 ml separating funnel. After adding 5 mL of bromocresol green solution and 2 mL of the buffer solution, the volume completed to 10 mL with distilled water. The aqueous solution was shacked for 10 sec and extracted three times using 5, 3 and 2 mL of chloroform. The organic phase was separated and dehydrated over about 1.0 g of anhydrous sodium sulfate and collected in a 10 mL volumetric flask. The flask was completed to volume with the same solvent. The absorbance of the resulting solution was measured at 415 nm against a reagent blank solution prepared by the same method.

*Optimization of the ion-pair complexation*

The effect of different variables on the formation of ion-pair complex, such as pH, time, extracting solvent and reagent concentration was studied by varying one parameter at a time and fixing other parameters. 

Also the stoichiometric relationship for complex formation was studied using Job's method of continuous variation (22). Solutions of tamsulosin hydrochloride (5 10^4^ M) and BCG (5 10^4^ M) were mixed using varying volume ratios with a fixed total volume of the mixture. The resulted absorbance at 415 nm was plotted against the mole fraction of tamsulosin to establish the molar ratio. 

*Method validation*

The linearity of the proposed method was tested using six series of tamsulosin hydrochloride standard solutions in the concentration range of 1-160 g/mL.

The precision and accuracy of the method was tested by analyzing three separate solutions of tamsulosin at 1, 40 and 160 g/mL in triplicate using the proposed method and constructed calibration curves in one day and three consecutive days.

*Analysis of pharmaceutical dosage form*

A commercial formulation containing 0.4 mg of tamsulosin hydrochloride (Omnic capsule) was analyzed using the proposed method. The sample preparation was according to a previously published method (23). The content of ten capsules were mixed and an amount of the granules equal to 0.8 mg of tamsulosin hydrochloride was accurately weighed and transferred into a 25 mL volumetric flask. After adding 12 mL of 0.05 M sodium hydroxide, the mixture sonicated for 30 min at 50 ºC. After addition of 10 mL of acetonitrile and sonicating for 5 min, 2 mL of 0.2 M hydrochloric acid was added and the solution sonicated again for 5 min. The pH was adjusted to 3.5 and the solution made up to volume by acetonitrile. The clear solution obtained after centrifugation at 3000 rpm for 10 min was analyzed using the proposed spectrophotometric method and also a reported HPLC method (24). The absorbance or the peak area was compared with a standard solution of tamsulosin prepared by the same procedure.

*Relative recovery*

To an amount of capsule granules equal to 2 capsules in a 25 mL volumetric flask, 2 mL of a standard solution of tamsulosin (400 g/mL) was added and the sample was processed according to the assay method. Percent recovery was calculated by comparing the absorbance of the spiked solution with a standard solution at the same concentration after subtracting the absorbance of the un-spiked sample.

## Results

*Absorption spectra*

Tamsulosin containing basic cationic nitrogen forms an ion-pair complex with BCG (an anionic dye) with a yellow color. The tamsulosin-BCG ion-pair complex extracted in chloroform showed maximum absorbance at 415 nm against reagent blank. This wavelength was used for all the experiments. The tamsulosin or BCG did not show significant absorbance in this region.

*Effect of pH and type of the buffer*

**Figure 2:**
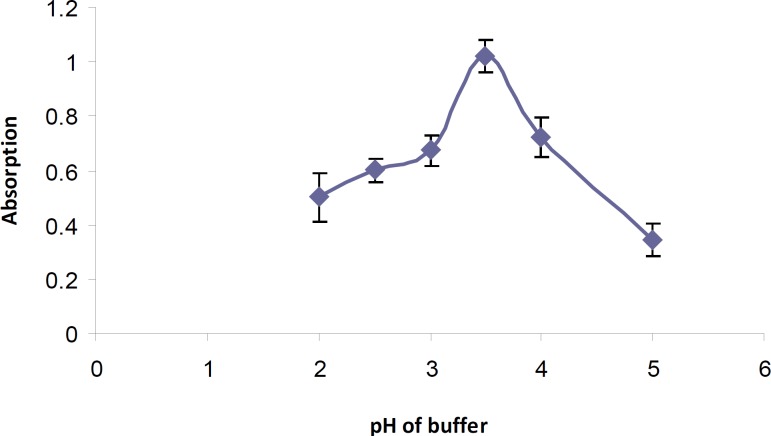
Effect of pH of Britton-Robinson buffer on tamsulosin-BCG ion-pair formation

The general procedure was performed using Britton-Rabinson buffer in the pH range of 2-5. The highest absorbance was observed at pH 3.5 (Figure 2). Different amounts of buffer were also added and maximum intensity was observed in the presence of 2 mL of buffer solution. 

Different buffer systems (phosphate, Britton-Robinson and phthalate) with the same pH values were tested and best results was achieved using phosphate buffer at pH 3.5.

It was also observed that the addition of buffer solution after the tamsulosin and BCG gave higher absorption values.

*Reagent concentration*

**Figure 3:**
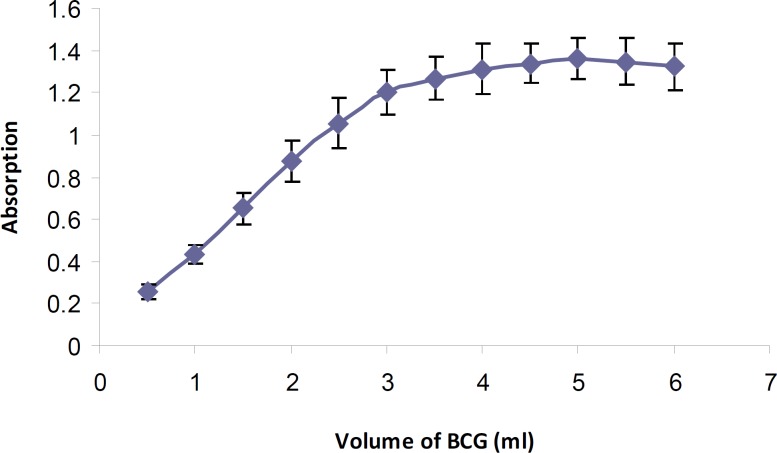
Effect of BCG volume (5 10^-4^ M) on tamsulosin-BCG ion-pair formation

Various amounts of BCG solution (0.5-6 mL) were added to a fixed concentration of tamsulosin hydrochloride according to the general procedure. Maximum absorbance intensity of the formed ion-pair complex was obtained using 5 mL of BCG solution (Figure 3). An immediate complex formation was developed at room temperature. Increasing the reaction time (0-60 min) had no effect on the absorbance intensity. Also the stability of the extracted ion-pair complex was studied for 24 h. The results showed that the resulted ion-pair complex was stable at least for 8 h (> 93%).

*Effect of extracting solvent*

Few organic solvents such as dichloromethane, chloroform, diethylether and ethylacetate were examined as extracting solvent. Maximum absorbance and also greater stability of the ion-pair complex and lower extraction of reagent blank was attained using chloroform as extracting solvent. It was also observed that three step extraction using 5, 3 and 2 mL of chloroform and 10 sec mixing for each step reached to better quantitative recovery of the ion-pair complex.

*Stoichiometry of the reaction*

**Figure 4:**
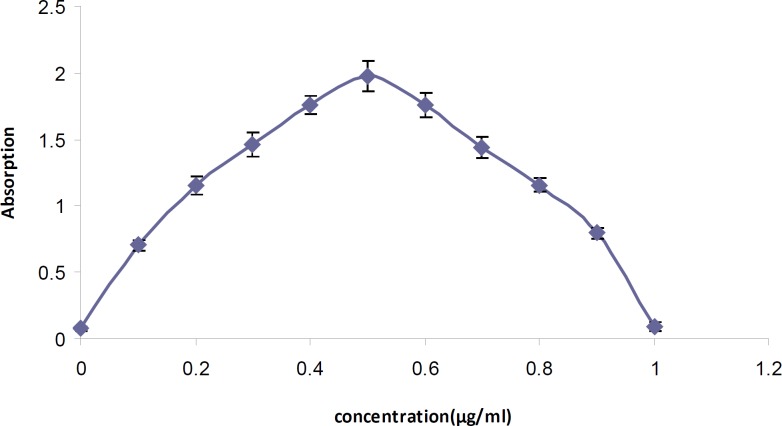
Continuous variation plot for the tamsulosin-BCG ion-pair complex using 5 10^-4^ M solutions.

The molar ratio of tamsulosin hydrochloride to BCG in the ion-pair complex in the reaction mixture according to the Job's method of continuous variations was indicated to be 1: 1 (Figure 4).

*Linearity*

The calibration curves constructed at eight concentration levels (1, 2, 5, 10, 20, 40, 80 and 160 g/mL) showed excellent linearity (R^2 ^> 0.999). The statistical data obtained are summarized in Table 1. The quantification limit and detection limit was calculated using the following equations (25):

LOQ= 10/s and LOD=3.3/s  

Where is the standard deviation of intercept and s is the slope of the calibration graph. According to the results obtained the LOQ and LOD was 1.1 and 0.37 g/mL respectively.

**Table 1:** Statistical data of the calibration curve of tamsulosin in standard solutions (n = 6)

**Parameters**	**Results**
Linearity range	μ
Regression equation	y = 0.0062 x - 0.0023
Standard deviation of slope	0.00008
Relative standard deviation of slope (%)	1.29
Standard deviation of intercept	0.0007
Correlation coefficient (r^2^)	0.9997

*Accuracy and precision*

The results of the accuracy and precision determination at three different concentration levels and three consecutive days are shown in Table 2. 

**Table 2:** Precision and accuracy of the method for determination of tamsulosin in standard solutions

**Concentration added** **(g/mL)**	**Within-day (n = 3)**			**Between-day (n = 9)**		
	**Found** **(g/mL)**	**CV (%)**	**Error (%)**	**Found** **(g/mL)**	**CV (%)**	**Error (%)**
1.00 40.00 100.00	0.980.03 39.890.43 161.960.21	3.06 1.08 0.13	-2.00 -0.28 1.23	0.990.03 39.450.60 161.181.09	3.03 1.52 0.68	-1.00 -1.38 0.74

*Relative recovery*

To find out the selectivity of the proposed method, the standard addition technique was applied. The relative recovery of tamsulosin hydrochloride from capsule granules was about 97% which shows no significant interferences from the excipients.

*Analysis of pharmaceutical formulation*

The developed method was carried out for determination of tamsulosin hydrochloride in Omnic capsules (0.4 mg). Comparable results were obtained by using a previously reported HPLC method in the literature (24) with no-significant difference (P < 0.05).

## Discussion

As mentioned previously, few spectrophotometric methods have been reported before for determination of tamsulosin hydrochloride in pharmaceutical dosage forms. In the method reported by Shrivastava *et al*. (14) bromocresol blue was used as complexing reagent. The reaction time was 2 min and the linearity range reported to be 2.5-22.5 µg/mL. In another method direct and derivative spectrophotometry were used for determination of tamsulosin hydrochloride (15). The linearity range was 10-90 µg/mL and the validation data were not completely studied. In the report of Gadhave *et al*. (16) also direct and derivative spectrophotometric methods were used and the linearity range was 1-6 µg/mL. The proposed method in this study showed linearity over a wide range of tamsulosin hydrochloride concentration. On the other hand the proposed method is relatively simple and time-consuming and showed suitable precision and accuracy to be used for quality control studies.

## References

[1] Goodman Brunton L, Parler K, Blumenthal D, Buxton I (2008). Goodman and Gillman's Manual of Pharmacology and Therapeutics.

[2] Macek J, Klima J, Ptacek P (2004). Rapid determination of tamsulosin in human plasma by high-performance liquid chromatography using extraction with butylacetate. J Chromatogr. B.

[3] Din L, Li L, Tao P, Yang J, Zhang Z (2002). Quantitation of tamsulosin in human plasma by liquid chromatography-electrospray ionization mass spectrometry. J Chromatogr. B.

[4] Ramakrishna NVS, Vishwattam KN, Manaj S, Koteshwara M, Wishu S, Vrama DP (2005). Rapid, simple and highly sensitive LC-ESI-MS/MS method for the quantification of tamsulosin in human plasma. Biomed Chromatogr.

[5] Matsushima H, Takanuki KI, Kamimura H, Watanabe T, Higuchi S (1997). Highly sensitive method for the determination of tamsulosin hydrochloride in human plasma dialysate, plasma and urine by high-performance liquid chromatography-electrospray tandem mass spectrometry. J Chromatogr. B.

[6] Keski-Rahkonen P, Parssinen O, Leppanen E, Mauriala T, Lehtonen M, Auriola S (2007). Determination of tamsulosin in human aqueous humor and serum by liqid chromtography-electrospray ionization tandem mass spectrometry. J Pharm. Biomed. Anal..

[7] Rao TSS, Tirumala R, Rao PS (2011). Quantification of tamsulosin in human plasma using LC-MS/MS. Bioanal Biomed..

[8] Chandrokar JG, Kotwal VB, Dhande NS, Gurav SG, Pande VV, Yadav PV (2008). A sensitive HPLC method for simultaneous estimation of tamsulosin hydrochloride and its impurity. Pak J. Pharm. Sci..

[9] Nithiyananthan TS, Shankarananth V, Rajasekhar KK, Ravikiran P, Vikram Kumar E, Jayanth Kumar Reddy G (2009). RP-HPLC method for the estimation of tamsulosin hydrochloride in bulk and tablet dosage form. Drug Invention Today.

[10] Nageswara Rao R, Kumar Talluri MVN, Narasa Raju A, Shinde DD, Ramanjareyulu GS (2008). Development of a validated RP-LCESI-MS/MS method for separation, identification and determination of related substances of tamsulosin in bulk drugs and formulations. J Pharm. Biomed. Anal..

[11] Choudhari VP, Nikalje APG (2009). Stability-indicating HPTLC method for the determination of tamsulosin in pharmaceutical dosage forms. Chromatographia.

[12] Zhang Z, Yang G, Linag G, Liu H, Chen Y (2004). Chiral separation of tamsulosin isomers by HPLC using cellulose tris (3, 5- dimethyl phenylcarbamate) as a chiral stationary phase. J Pharm. Biomed. Anal..

[13] Maier V, Horakova J, Petr J, Tesarova E, Coufal P, Sevcik J (2005). Chiral separation of tamsulosin by capillary electrophoresis. J Pharm. Biomed. Anal..

[14] Shrivastava A, Saxena P, Gupta VB (2011). Spectrophotometric estimation of tamsulosin hydrochloride by acid-dye method. Pharm Methods.

[15] Bari SB, Bakshi AR, Jain PS, Surana SJ (2011). Application of UV-spectroscopy and first order derivative method for determination of tamsulosin hydrochloride in bulk and tablets. Pharm Anal. Acta.

[16] Gadhave NA, Sawant SD, Ghante MR, Nikam AD (2011). Spectrophotometric estimation of tamsulosin hydrochloride in tablet dosage form. Int J. Pharm. Res. Dev..

[17] Amanlou M, Hoseinzadeh Nazlou M, Azizian H, Souri E, Farsam H (2007). Determination of ketotifen fumarate in raw material and pharmaceutical products using ion-pair formation. Anal Lett..

[18] Amanlou M, Souri E, Izady Sh, Farsam H (2007). Spectrophotometric determination of tropicamide in bulk and pharmaceutical formulatuins. Iran J. Pharm. Res..

[19] Amanlou M, Keivani S, Sadri B, Gorban-dadras O, Souri E (2009). Simple extractive colorimetric determination of buspirone by acid-dye complexation method in solid dosage form. Res Pharm. Sci..

[20] Shamsa F, Amani L (2006). Determination of sulfamethoxazole and trimethoprim in pharmaceuticals by visible and UV spectrophotometry. Iran J. Pharm. Res..

[21] Kazemipour M, Ansari M (2005). Derivative spectrophotometry for simultaneous analysis of chlorpheniramine maleate, phenylephrine HCl, and phenypropanolamine HCl in ternary mixtures and pharmaceutical dosage forms. Iran J. Pharm. Res..

[22] Huang CY (1982). Determination of binding stoichiometry by the continuous variation method. The Job Plot. Methods Enzymol.

[23] Basaveswara Rao MV, Reddy BSK, Subba Rao M, Sreedhar B (2009). Development and validation of RP-HPLC method for the determination of tamsulosin hydrochloride. Int J. Chem. Sci..

[24] Nithiyananthan TS, Shankarananth V, Rajasekhar KK, Hareesh G, Naveen Kumar P, Siva Prasada Reddy R (2009). Formulation and evaluation of tamsulosin hydrochloride as sustained release matrix tablet. Int J. Chem. Tech. Res..

[25] Shabir GA (2003). Validation of high-performance liquid chromatography methods for pharmaceutical analysis. Understanding the differences and similarities between validation requirements of the US Food and Drug Administration, The US Pharmacopeia and the International Conference on Harmonization. J. Chromatogr. A.

